# The One-Step Pickering Emulsion Polymerization Route for Synthesizing Organic-Inorganic Nanocomposite Particles

**DOI:** 10.3390/ma3021186

**Published:** 2010-02-16

**Authors:** Huan Ma, Mingxiang Luo, Sriya Sanyal, Kaushal Rege, Lenore L. Dai

**Affiliations:** Chemical Engineering, Arizona State University, Tempe, AZ 85287, USA; E-Mails: huan.ma@asu.edu (H.M.); mluo5@jhu.edu (M.L.); sriya.sanyal@asu.edu (S.S.); kaushal.rege@asu.edu (K.R.)

**Keywords:** Pickering emulsion polymerization, nanocomposite particles, temperature sensitivity

## Abstract

Polystyrene-silica core-shell nanocomposite particles are successfully prepared *via* one-step Pickering emulsion polymerization. Possible mechanisms of Pickering emulsion polymerization are addressed in the synthesis of polystyrene-silica nanocomposite particles using 2,2-azobis(2-methyl-*N*-(2-hydroxyethyl)propionamide (VA-086) and potassium persulfate (KPS) as the initiator. Motivated by potential applications of “smart” composite particles in controlled drug delivery, the one-step Pickering emulsion polymerization route is further applied to synthesize polystyrene/poly(N-isopropylacrylamide) (PNIPAAm)-silica core-shell nanoparticles with N-isopropylacrylamide incorporated into the core as a co-monomer. The polystyrene/PNIPAAm-silica composite nanoparticles are temperature sensitive and can be taken up by human prostate cancer (PC3-PSMA) cells.

## 1. Introduction

Solid particles have been identified as a new type of emulsifying agent in addition to surfactants and amphiphilic polymers since the pioneering studies by Ramsden in 1903 [1] and Pickering in 1907 [2]. Such emulsions are later on named as Pickering emulsions. In Pickering emulsions, solid particles of intermediate wettability in the size range from several nanometers to several micrometers attach to liquid-liquid interfaces and provide emulsion stability. Recently, there has been growing interest in Pickering emulsions because they open new avenues of emulsion stabilization and have numerous practical applications. For instance, we have studied the fundamentals of Pickering emulsions [3,4], utilized them as templates to investigate the dynamics of particles [5,6], and developed microrheology at liquid-liquid interfaces [7,8,9]. In this report, we further apply the concept of Pickering emulsions to synthesize core-shell nanocomposite particles.

It is worthwhile to note that the nanocomposite particle structure in this study is opposite to the often reported core-shell structure in which inorganic particles serve as the core and polymer serves as the shell [10,11,12,13,14,15]; here the polymer serves as the core and the inorganic particles serve as the shell. Such materials provide a new class of supramolecular building blocks and can “exhibit unusual, possibly unique, properties which cannot be obtained simply by co-mixing polymer and inorganic particles.” [16] In comparison with the recently reported methods to synthesize core-shell composite particles, such as post-surface-reaction [17,18], electrostatic deposition [19], and layer-by-layer self-assembly [11,20,21], Pickering emulsion polymerization is superior in several aspects: (1) no sophisticated instrumentation is needed; (2) a commercialized nanoparticle powder or solution can be used without further treatment; (3) the synthesis can be completed in one-step; and (4) the produced particle dispersion is surfactant-free. Despite these advantages, efforts to explore and utilize this approach is limited, although pioneer explorations have been initiated on some approaches including miniemulsion polymerization [22,23], dispersion polymerization [24,25,26], inverse suspension polymerization [27,28], and inverse emulsion polymerization [29] stabilized by fine solid particles. During the progress of this work, Lee and coworkers [30] successfully synthesized polystyrene-silica composite particles using Pickering emulsion polymerization; however, the silica nanoparticles need to be added 30 minutes after the initiation which complicated the process. In this work, we report the successful synthesis of polystyrene-silica core-shell nanocomposite particles and polystyrene/poly(N-isopropylacrylamide) (PNIPAAm)-silica core-shell nanoparticles *via* one-step Pickering emulsion polymerization, address the mechanisms of Pickering emulsion polymerization, and finally, investigate the cellular uptake of the polystyrene/PNIPAAm-silica core-shell nanoparticles. 

## 2. Results and Discussion

### 2.1. Synthesis and Characterizations of Nanocomposite Particles

**Figure 1:**
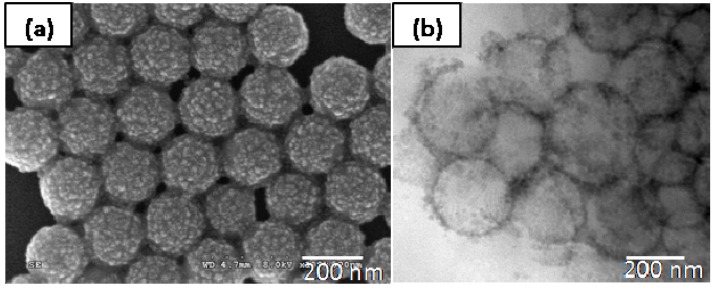
An SEM image **(a)** of the nanocomposite particles prepared using VA-086 as the initiator and a TEM image **(b)** of cross-sectioned composite particles.

**Figure 2:**
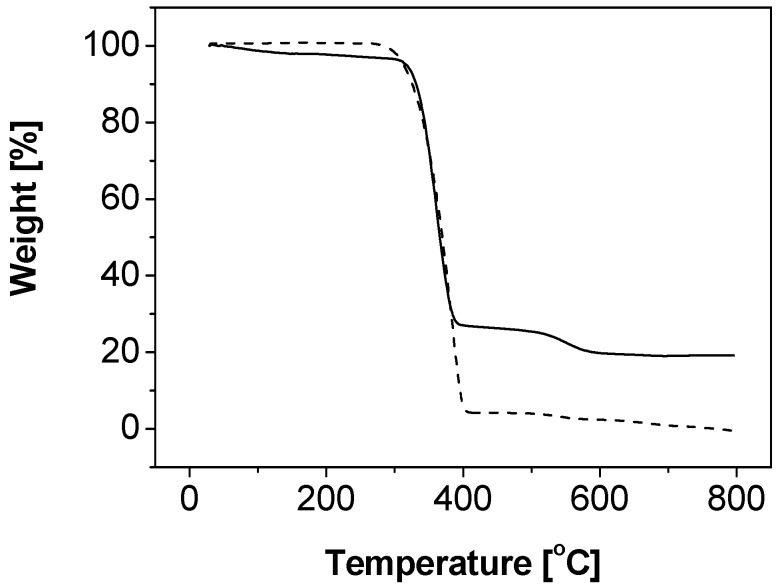
Thermogravimetric analysis of the nanocomposite particles prepared using VA-086 as the initiator before (solid line) and after (dashed line) HF etching treatment.

Polystyrene-silica nanocomposite particles were successfully prepared by formulating 8 mL styrene, 52.5 mL water, 20 g IPA-ST silica nanoparticle dispersion (10–15 nm silica nanoparticles dispersed in isopropanol at the concentration 30–31 wt %), and 0.06 g initiator 2,2-azobis(2-methyl-*N*-(2-hydroxyethyl)propionamide (VA-086). The particle size, characterized by dynamic light scattering (DLS), is 203.9 ± 51.6 nm in diameter with mono-distribution (the standard deviation here represents the half width of the particle size distribution). Figure 1(a) is a representative SEM image of the composite particles sampled at 5 hour reaction time. The roughness of the composite particle surfaces suggests that the composite particles are covered by silica nanoparticles. The core-shell structure can be clearly observed in the TEM image presented in Figure 1(b). In many regions, the thickness of the shell is close to the size of one silica nanoparticle (10–15 nm), which may suggest monolayer coverage. The silica content is quantitatively determined by TGA, as shown in Figure 2 (solid line). Two samples were measured: the composite particles (solid line) and the composite particles after removal of the silica component by hydrofluoric acid etching, which is essentially polystyrene cores (dashed line). The polystyrene cores show a residual weight of approximately zero at 800 °C. Thus it is reasonable to assume that the major weight loss during heating is associated with the thermo-oxidative degradation of polystyrene and the residue close to 800 °C is solely silica. The silica content of the composite particles is approximately 20 wt %. Although some silica nanoparticles remain in the continuous phase and are washed off by centrifuging-redispersing cycles, the silica content of particles prepared *via* solid-stabilized emulsion polymerization using nonionic initiator VA-086 is significantly higher than that of particles (1.1 wt %) prepared *via* dispersion polymerization using nonionic initiator AIBN [24]. The improvement is likely due to the distinct polymerization mechanisms. In contrast to the dispersion polymerization in which the polystyrene monomers are dissolved in alcohols, the emulsion polymerization here contains distinguishable liquid-liquid interfaces due to the immiscibility between the monomers and the aqueous continuous phase. Therefore the nanoparticles, even in the absence of electrostatic interactions, are thermodynamically favorable to self-assemble and remain at the liquid-liquid interfaces, following the same argument in Pickering emulsions [3,4,31,32]. At the initial stage of polymerization, the nanoparticles provide stability to the monomer droplets. During the nucleation stage, silica nanoparticles are at the interface between the monomer phase and continuous phase. It is worthwhile to note that the role of silica nanoparticles described here is not the same as that in the polymerization involving oppositely charged initiator and nanoparticles [24]. In the latter case, the initiator molecules or residues adsorb onto the silica nanoparticle surfaces after initiation [24] thus the silica nanoparticles function as the surface-active initiator residue. The mechanism of the core-shell structure formation in Pickering emulsion polymerization will be detailed later on.

In the above experiments, we carefully selected VA-086 as the initiator. VA-086 is a water-soluble nonionic initiator and no success has been reported in surfactant-free emulsion polymerization of styrene [33]. In order to verify the sole stabilizing effect of silica nanoparticles, emulsifier-free emulsion polymerization using VA-086 as the initiator in the absence of nanoparticles was performed. No polystyrene particle formation was observed in the product, evidenced by SEM experiments. These experiments show that the initiator VA-086 has little effect on stabilizing the system in emulsion polymerization and therefore silica nanoparticles here are the only source of stabilizer. In addition, VA-086 is neutral in charge thus is expected to minimize any electrostatic interactions with the negatively-charged silica nanoparticle surfaces. 

**Figure 3:**
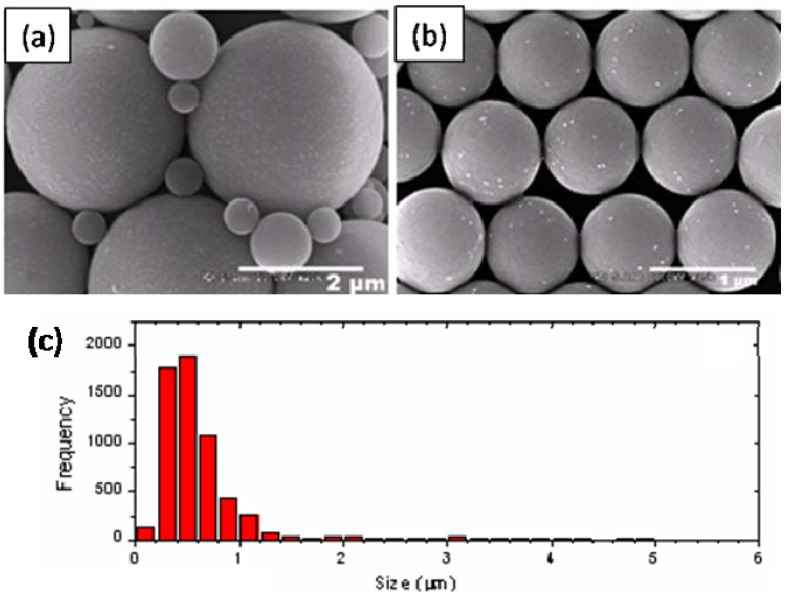
SEM images **(a-b)** and the size distribution **(c)** of polystyrene-silica particles prepared using KPS as the initiator.

**Figure 4:**
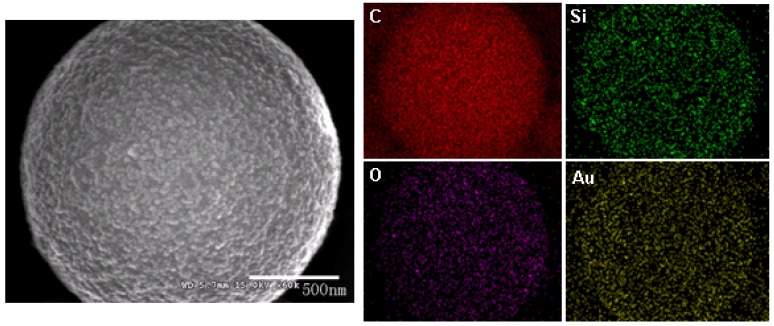
The SEM image and energy dispersive X-ray spectrometry analysis element mapping of a polystyrene-silica particle prepared using KPS as the initiator. Red, green, purple and gold represent carbon, silicon, oxygen, and gold elements, respectively.

The effect of initiator type on Pickering emulsion polymerization is studied by changing the initiator to potassium persulfate (KPS). KPS is a water-soluble anionic initiator and has been shown to provide sufficient stabilizing effect during surfactant-free emulsion polymerization [34,35]. Figure 3 shows the SEM images and size distribution of composite particles prepared using 0.06 g initiator KPS, 16 mL styrene, 52.5 mL water, 8 g IPA-ST, and 0.6 g sodium bicarbonate. The particle size is mostly several hundred nanometers although particles of several micrometers are also present. Interestingly, only the micron-sized particles are covered with silica nanoparticles whereas the sub-micron-sized particles have little silica coverage. This may suggest the existence of different particle formation mechanisms (see Section 2.2). To analyze the chemical composition and distribution, energy dispersive x-ray spectrometry analysis was performed on micron-sized particles and a sample element mapping is presented in Figure 4. Carbon element is concentrated on the particles; silicon and oxygen are also observed, which confirm the presence of silica. The gold element comes from the sputter coating process during SEM sample preparation. 

### 2.2. Mechanisms of Pickering Emulsion Polymerization

The mechanism of conventional emulsion polymerization stabilized by surfactants has been under active discussion for over half a century and some consensus has been reached. Harkins proposed three loci of particle nucleation in 1947 [36], which are later developed into at least three different nucleation mechanisms [37]: the micellar nucleation, the homogeneous coagulative nucleation, and the droplet nucleation. Upon initiator addition and decomposition, free radicals form in the aqueous phase. The micellar nucleation [37,38] begins with the capture of free radicals by micelles, proceeds with the continuous swelling and polymerization of monomers in the monomer-swollen particles, and finally terminates with the exhaustion of monomers. While some researchers believe that the micellar nucleation mechanism dominates at a surfactant concentration above the critical micelle concentration, doubts have also been raised [38]. In the absence of micelles, the homogeneous coagulative nucleation mechanism is likely dominant. In homogeneous coagulative nucleation [37,39,40,41,42], monomers dissolve in water and undergo radical polymerization to form oligomers. The oligomers coagulate to form embryos, nuclei, and primary particles sequentially. These primary particles, stabilized by the adsorption of surfactant molecules, could grow either *via* swelling of particles by monomers or deposition of oligomers onto their surfaces [43]. Finally, droplet nucleation is another possible mechanism in conventional emulsion polymerization. Here the monomer droplets may be subjected to the oligomeric radical entry and solidify into particles, following the droplet nucleation mechanism. Droplet nucleation is usually minor in emulsion polymerization, except in miniemulsion polymerization when hydrophobic initiators are used. 

**Figure 5:**
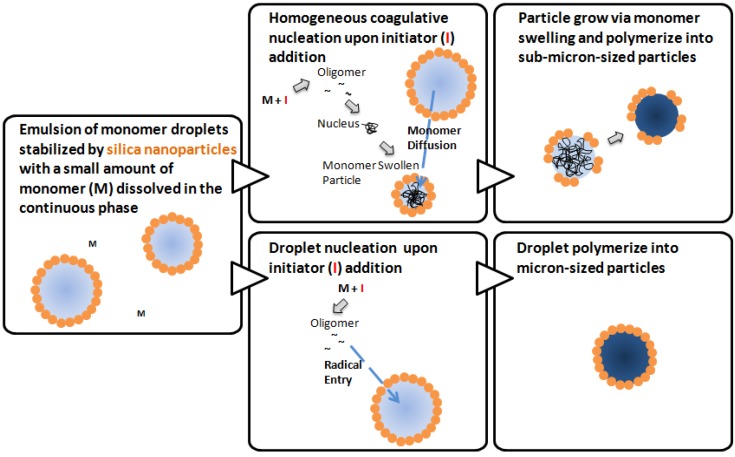
Schematic illustration for possible mechanisms of Pickering emulsion polymerization.

Based on the fundamental understandings in conventional emulsion polymerization, we propose possible Pickering emulsion polymerization mechanisms, taking into account the differences between fine solid particles and surfactant molecules. Since the nanoparticles do not form micelles like surfactant molecules, micellar nucleation is excluded. Thus, there are two possible nucleation mechanisms involved in the initial stage of Pickering emulsion polymerization. Homogeneous coagulative nucleation is likely to be the dominating mechanism here, which yields the sub-micron-sized particles. The droplet nucleation might also occur, which yields micron-sized particles. The two mechanisms are illustrated in Figure 5. Upon initiator addition, monomers dissolved in the aqueous phase react with decomposed initiators and form oligomers with radicals. In homogeneous coagulative nucleation, the oligomers coagulate into nuclei, which subsequently become monomer swollen particles. Nanoparticles self-assemble at the interfaces between monomer and the continuous phase to provide stability. With the continuous supply of monomer molecules from the monomer droplets through diffusion, the particle size growth is mainly achieved by monomer swelling followed by polymerization within the core. In contrast, in droplet nucleation, initiated oligomers with radicals enter monomer droplets and subsequently polymerize into solid cores without significant size growth. 

**Figure 6:**
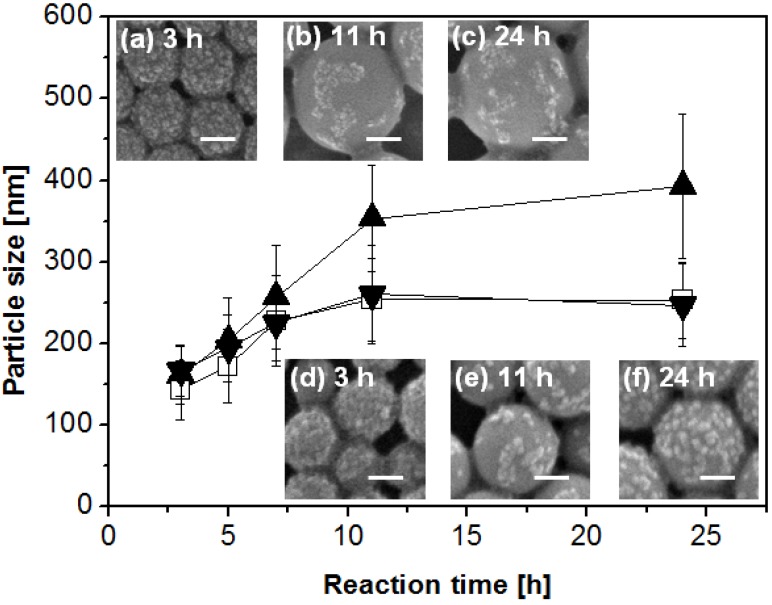
Plot of particle size *versus* reaction time and representative SEM images with different initiator VA-086 concentrations: 0.83 wt % (▲, inset images a, b and c), 2.5 wt % (□) and 4.2 wt % (▼, inset images d, e and f). The error bars indicate the width of the particle size distribution and the scale bars represent 100 nm.

First, we use the hypothesized mechanisms to interpret the formation of polystyrene-silica nanocomposite particles prepared using VA-086 as the initiator. Figure 6 shows the dependence of particle size and surface coverage on reaction time and initiator concentration [44]. The nanocomposite particles are sampled from 3 h to 24 h reaction time and the initiator concentration relative to monomer is selected to be 0.83, 2.5, and 4.2 wt % respectively. At 3 h reaction time, well after the nucleation stage, nanocomposite particles with dense silica coverage are obtained. Since VA-086 initiator residues cannot provide sufficient stabilization to the monomer-swollen particles, silica nanoparticles would self-assemble at interfaces to provide stabilization and thus lead to high silica coverage. At initiator concentration 0.83 wt %, the silica coverage decreases with the particle size growth and the silica nanoparticles form patches on the nanocomposite particle surface with a low coverage. This might be an indication that the surface area of the polystyrene core increases with the particle growth without a significant increase of silica continuously attaching onto the polystyrene core. The particle growth mechanism is likely due to swelling of particles by monomers in the continuous phase. The same mechanism explains the surface coverage decrease in the system containing 2.5 wt % of initiator (images not shown) and from 3 h to 11 h in the system containing 4.2 wt % of initiator. These observations suggest that the Pickering emulsion polymerization using VA-086 as the initiator mainly follows the homogeneous coagulative nucleation mechanism. 

One remaining mystery is the unexpected silica coverage from 11 h to 24 h in the system with 4.2 wt % initiator. Although the origin is unclear, we tentatively attribute the unusual silica coverage increase to the deposition of oligomers on the polystyrene core [43], which adsorbed onto silica nanoparticles in the continuous phase [43]. Excess initiator molecules might generate a large number of oligomers in the continuous phase, which could possibly adsorb onto silica nanoparticles. Thus when the oligomers on silica nanoparticles attach to preformed polystyrene surfaces, the silica nanoparticles are anchored there. It is also possible that the surface coverage increase might be due to the adsorption of depleted or close to depleted monomer droplets with a size below that of particles. It is also worthwhile to note that the continuous phase contains approximately 21% isopropanol. The existence of isopropanol might increase the solubility of the monomer and the degree of polymerization required for an oligomer to be insoluble in the continuous phase, however, the solubility of monomer in the continuous phase is still low enough to enable emulsification and subsequent emulsion polymerization.

**Figure 7:**
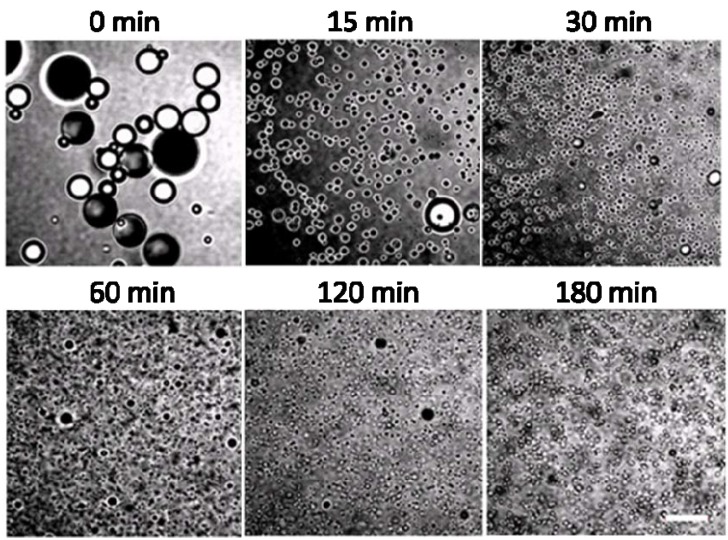
Representative transmitted light images viewed on a confocal microscope of the polymerization system sampled at different time intervals after initiation. The scale bar represents 20 µm.

The mechanism appears to be more complicated when KPS is used as the initiator, likely due to the charged initiator residue which has repulsive interactions with the similarly charged nanoparticles and the stabilizing effect provided by the initiator residue. With an objective to understand the origins of the high-silica-coverage on micron-sized particles and the low-silica-coverage on sub-micron-sized particles, we emphasize the initial stage of polymerization. Figure 7 shows representative transmitted light images observed *via* a confocal microscope of the emulsion droplets/particles sampled at different time intervals during the synthesis. At the time of initiator addition, the emulsion droplet size is primarily determined by the emulsification process and we observe a wide size distribution of emulsion droplets from 1 to 20 μm. The emulsion droplets are mostly covered by silica nanoparticles. The droplets appear in black when focusing at the silica covered upper or lower interface, but show a black ring when the focus plane is the droplet edge. Within 30 minutes after initiator addition, the size of majority of the emulsion droplets significantly decreases. Nucleation takes place in the continuous phase within this period of time. Following the homogeneous coagulative nucleation mechanism [40,41,42], monomer molecules initially dissolved in the aqueous phase form oligomers. Subsequently, the oligomers coagulate into nuclei stabilized by negatively charged hydrophilic initiator residue groups and further grow into monomer swollen particles. The negative charge at the interface results in a low silica coverage at the interface due to the repulsion between initiator residue and silica nanoparticles. Through monomer swelling and polymerization, sub-micron-sized particles with low silica coverage are obtained. A close examination of the images taken from 30 to 180 minutes reveals the presence of a small population of micron-sized monomer droplets or particles which are likely to follow the droplet nucleation mechanism. The micron-sized droplets could possibly be subjected to radical entry and polymerize into micron-sized particles, although charged nanoparticles at the interface may act as a barrier to radical entry. The silica nanoparticles initially adsorbed at droplet interfaces remain at the interface during the polymerization as the droplets solidify, which leads to a high silica coverage of these composite particles. 

### 2.3. Potential Applications of Composite Nanoparticles in Controlled Drug Delivery

Organic-inorganic composites are ubiquitous in biological and medical applications such as in artificial bones, dental fillings, and drug delivery [45]. Among them, core-shell composite nanoparticles are a unique class of materials and open tremendous new opportunities for targeted drug delivery. The main objective for targeted and controlled drug delivery is to minimize or eliminate undesirable side effects and deliver therapeutics in a reproducible manner to a specific target at the required level. Although drug delivery has been an active research topic for more than a century, there remain critical challenges for targeted and controlled drug delivery in terms of effective synthesis, release rate, and release time. PNIPAAm is a well-understood temperature sensitive gel, which undergoes volume shrinkage at a transition temperature of approximately 32 °C in pure water [46]. The mechanism of this change is based on different solubility below and above the lower critical solution temperature (LCST) in aqueous media. Below the LCST, the polymer chain is hydrophilic as the hydrogen bonding between the hydrophilic groups and water molecules dominates; above the LCST, the polymer chain becomes hydrophobic due to the weakened hydrogen bonding at elevated temperature and the hydrophobic interactions among hydrophobic groups [47]. Importantly for triggered drug delivery *in vivo*, PNIPAAm can be engineered to possess temperature slightly above physiological temperatures (e.g., at 38–39 °C) [48], which can be exploited for selective and controlled delivery at the tumor site using external stimuli (e.g. laser irradiation).

**Figure 8:**
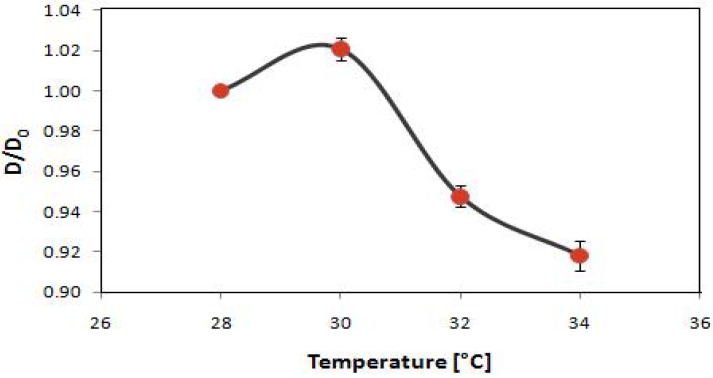
The dependence of normalized average diameter of the composite nanoparticles on temperature. The initial diameter at 28 °C for each individual batch is used for normalization. The error bars show standard deviations of particles made in three different batches and the curve smoothly connects the data points.

Motivated by potential applications of “smart” composite particles, PNIPAAm was incorporated into the core of the nanocomposite. Pickering emulsion polymerization of the NIPAAm/styrene monomer mixture (NIPAAm concentration 15%) was performed using VA-086 as the initiator. An apolar dye, BODIPY 493/503, was loaded in the core for fluorescent imaging and as a model drug. The polystyrene/PNIPAAm-silica core-shell nanoparticle is responsive to thermal stimuli. Figure 8 shows the dependence of normalized average diameter of the composite particles on temperature measured with DLS. The average particle size at 28 °C (D_0_) is approximately 71 nm. The size increases slightly when the temperature reaches 30 °C and then decreases sharply as heated across 32 °C, around the LCST for homopolymer PNIPAAm. The transition temperature is not shifted by the silica nanoparticle encapsulation. This is consistent with the recently reported composite microspheres with a PNIPAAm core and a silica shell which also show a volume transition starting at 32 °C [27]. The reason given by the authors is that silica particles are physically adsorbed on the surfaces of PNIPAAm microspheres. Moreover, the copolymerization with styrene has no significant effect on the transition temperature. One hypothesis is the phase separation of PNIPAAm and polystyrene within the core, supported by a study on PNIPAAm-styrene copolymer particles which suggests a PNIPAAm-rich shell and a polystyrene-rich core structure [49]. 

**Figure 9:**
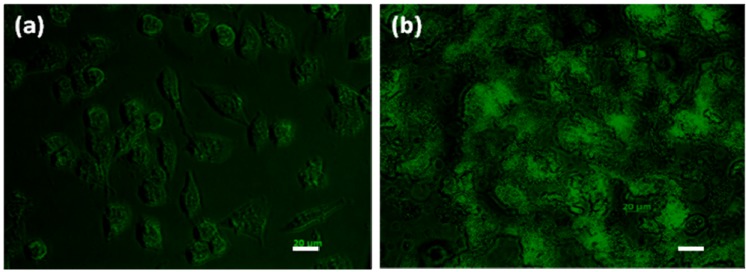
Cellular uptake of polystyrene/PNIPAAm core-silica shell nanoparticles by PC3-PSMA prostate cancer cells at low **(a)** or high **(b)** nanoparticle dosage. Scale bars represent 20 µm.

It was hypothesized that polystyrene/PNIPAAm-silica core-shell nanoparticles are sufficiently small to be taken up by cancer cells. Cell uptake experiments were performed using PC3-PSMA human prostate cancer cells (50,000/well in 24 well plates). The uptake of the composite nanoparticles by the cells with incubation time of 5 hours was visualized using fluorescence microscopy. Figure 9 shows the fluorescence images of cells after exposure to the composite nanoparticles at different dosages. At low nanoparticle dosage, the PC3-PSMA human prostate cancer cells take up dye-loaded composite nanoparticles and traffic them to different intracellular vesicles throughout the cytoplasm while remaining viable. Figure 9a suggests that composite nanoparticles are internalized in the cells. Nanoparticles could be transported into the cell by either specific or nonspecific cellular uptake mechanisms depending on the surface properties. Since the nanoparticles are not conjugated with any antibody, the uptake behavior here is nonspecific. In addition, the nonspecific internalization might be due to the affinity between clathrin coated vesicles and silica nanoparticle coated outer shell and the lack of any specific ligands for receptor-mediated endocytosis [50]. Nonspecific uptake of nanoparticles has recently attracted much attention in the development of new strategies for designing efficient nano-carriers, though specific uptake is a more developed strategy as more control is possible and effects on cell functions are easier to predict. Higher nanoparticle dosage could destroy the integrity of the cell and make the cells non-viable, as shown in Figure 9b. Efforts are ongoing to quantitatively determine the maximum particle dosage for the cells to remain viable and the dependence of uptake amount on the incubation time and temperature. This preliminary study demonstrates that the thermal sensitive composite nanoparticles can be taken up by PC3-PSMA cells and opens new opportunities in controlled drug delivery.

## 3. Experimental Section

### 3.1. Materials

IPA-ST (Nissan Chemicals) is a dispersion of 10–15 nm silica nanoparticles in 2-isopropanol at a concentration of 30–31 wt %. Nonionic azo initiator VA-086 (98%, 2,2-azobis(2-methyl-*N*-(2-hydroxyethyl)propionamide), Wako Chemicals), anionic initiator potassium persulfate (KPS, 99%, Acros Organics), styrene monomer (99.9%, Fisher), N-isopropylacrylamide monomer (NIPAAm, 97%, Aldrich), and water (HPLC grade, Acro Organics) were used in the polymerization without further purification. The nonpolar dye BODIPY with excitation/emission maxima being approximately 493/503 nm (4,4-difluoro-1,3,5,7,8-pentamethyl-4-bora-3a,4a-diaza-s-indacene) was obtained from Invitrogen, Molecular Probes. The serum-free medium contains RPMI-1640 medium, 25 mM HEPE, L-Glutamine, 1% Penicillin/streptomycin (HyClone, UT). The serum-containing medium has 10% FBS (Heat inactivated fetal bovine serum, defined SH30071.03, HyClone, UT) in addition to the serum-free medium. Phosphate buffered saline (PBS) was formulated as 0.14 M NaCl, 10 mM Na_2_HPO_4_, and 2.7 mM KCl, and the pH was adjusted to 7.4 with HCl and NaOH. PC3-PSMA cells were generous gifts from Dr. Michael Sadelain, Memorial Sloan Kettering Cancer Center, New York. The PC3-PSMA cell line is a sub-clone of PC3 cells retrovirally transduced to stably express the PSMA receptor [51]. All cells were grown in RPMI-1640 (HyClone, UT) containing 10% fetal bovine serum (HyClone, UT) and 1% penicillin/streptomycin (HyClone, UT) in 5% CO_2_ at 37 °C in an incubator.

### 3.2. Composite Particle/Nanoparticle Synthesis

The composite particles were prepared using the following procedure. First, water, IPA-ST and styrene were agitated mechanically at 600 rpm for 8 min using Arrow 6000 (Arrow Engineering) in an ice bath to emulsify. Second, the emulsion was degassed with nitrogen and kept in nitrogen atmosphere under magnetic stirring. When the temperature was raised to 70 °C, the initiator aqueous solution was added to start the polymerization. The composite particles were sampled at different time intervals ranging from 3 h to 24 h. A typical formulation includes 8 mL styrene, 52.5 mL water, 20 g IPA-ST silica nanoparticle dispersion, and 0.06 g initiator VA-086. In the synthesis using KPS as the initiator, sodium bicarbonate was added into water prior to emulsification. A typical formulation includes 16 mL styrene, 52.5 mL water, 8 g IPA-ST, 0.06 g initiator KPS, and 0.6 g sodium bicarbonate. In the synthesis of thermal sensitive composite nanoparticles, emulsification was performed with an IKA Ultra Turrax T25 homogenizer at 10,800 rpm for 2 minutes in an ice bath. The dye BODIPY 493/503 was added after the emulsification stage prior to adding the initiator. A typical formulation includes 0.66 g NIPAAm, 3.76 g styrene, 32 mL water, 4.1 g IPA-ST silica nanoparticle dispersion, 0.037 g initiator VA-086, and 1 µg BODIPY 493/503.

Before characterization and uptake experiments, the samples were washed twice by centrifuging-redispersing cycles using an Eppendorf 5810R centrifuge. In each cycle, the sample was centrifuged at 7,000 rpm for 5 minutes, the supernatant was replaced with water and the sediment was redispersed by shaking manually.

### 3.3. Hydrofluoric Acid Etching

To remove the silica shell, the HF etching procedure described by Han and coworkers [52] was adopted. Approximately 1 mL of original composite particle dispersion was added to 50 mL 10% HF aqueous solution. Then the mixture was stirred at room temperature (approximately 23 °C) for 2 hours. After settling for several hours, the supernatant was replaced with water for at least three times and the sediments were obtained.

### 3.4. Determination of Particle Size

Particle size and distribution of composite particles prepared using VA-086 as the initiator were measured using a Microtrac Nanotrac or Brookhaven 90Plus Particle Size Analyzer with the dynamic light scattering (DLS) technique. The washed composite particles were further dispersed to proper concentrations with water before measurements. The particle size and distribution of composite particles prepared using KPS as the initiator were determined from SEM images using the Image J software.

### 3.5. Particle Morphology and Composition Characterization

A scanning electron microscope (SEM, Hitachi S-4300) was used to observe the surface morphology of the composite particles. SEM samples were prepared by drying a drop of washed composite particle dispersion on newly cleaved mica substrate, and applying Au/Pd coating of 10–15 nm thick using a sputter coater. Energy dispersive X-ray spectrometry (EDX), built in the S-4300 SEM, was used to qualitatively determine the chemical composition and obtain the element mapping of the composite particles. In EDX analysis, pure carbon substrate was selected. To quantitatively characterize the silica content of the particles, thermogravimetric analysis (TGA) was performed using Mettler-Toledo SDTA851e. Samples were heated to 800 °C at 10 °C/min in air. In order to image the cross-sectioned composite particles, the particles were embedded in a LX-1122 epoxy resin block and cut into 90–150 nm sections using a Reichert-Jung UltraCut E microtome equipped with a glass knife at room temperature. The thin sections were collected with copper grids and observed using the transmission electron microscope (TEM, Hitachi H-8100).

### 3.6. Confocal Microscope Observations of the Sampled Mixtures during Polymerization

The polymerization mixture was sampled at different time intervals from 0 to 180 minutes. The samples were immediately diluted and observed using an Olympus FV300 laser scanning confocal microscope under transmitted light mode. 

### 3.7. Cellular Uptake Experiments

Polystyrene/PNIPAAm-silica core-shell nanoparticles loaded with BODIPY 493/503 dye in the core for imaging and as a model drug, were used in cellular uptake experiments. First, PC3-PSMA prostate cancer cells were plated in a 24 well plate (50,000 cells per well) and incubated for 16 h at 37 °C. PC3-PSMA cells were placed at 37 °C in an incubator with dye-containing nanoparticles for 5 h in the presence of serum-free medium, followed by extensive washing with PBS. Then, serum containing medium was added and the cells incubated for 20 minutes. Finally, the cells were fixed and imaged using phase contrast and fluorescence using a Zeiss Axiovert 200 microscope. 

## 4. Conclusions

In summary, nanocomposite particles with polystyrene core and silica shell were successfully synthesized *via* one-step Pickering emulsion polymerization using silica nanoparticles as the stabilizers. In the polymerization formulated with 8 mL styrene, 52.5 mL water, 20 g IPA-ST, and 0.06 g initiator VA-086, the nanocomposite particles sampled at 5 hour reaction time exhibit core-shell structure in which the polystyrene core is covered by silica. The nanocomposite particles are 203.9 ± 51.6 nm in diameter with a silica content of approximately 20 wt %. When KPS is used as the initiator, the particle size distribution becomes broader with the existence of micron-sized particles. The sub-micron-sized particles are barely covered with silica nanoparticles, likely due to the repulsive interaction between the anionic initiator residues and the negatively charged nanoparticles. Possible mechanisms of Pickering emulsion polymerization are also discussed. Homogeneous coagulative nucleation is identified as the dominating mechanism in polymerization when using VA-086 as the initiator whereas both homogeneous coagulative nucleation and droplet nucleation exist in the system with KPS. Finally, polystyrene/PNIPAAm-silica core-shell nanoparticles were successfully synthesized with N-isopropylacrylamide incorporated into the core of the nanocomposite as a co-monomer at a concentration of 15%. The composite nanoparticles are temperature sensitive and can be taken up by PC3-PSMA human prostate cancer cells, which indicates the potential application as “smart” and controlled drug delivery vehicles.
